# Plasma protein patterns are strongly correlated with pressure pain thresholds in women with chronic widespread pain and in healthy controls—an exploratory case-control study

**DOI:** 10.1097/MD.0000000000020497

**Published:** 2020-05-29

**Authors:** Björn Gerdle, Karin Wåhlén, Bijar Ghafouri

**Affiliations:** Pain and Rehabilitation Centre, and Department of Health, Medicine and Caring Sciences, Linköping University, Linköping, Sweden.

**Keywords:** biomarker, fibromyalgia, pain, pressure pain threshold, sensitivity, widespread pain

## Abstract

Chronic widespread pain (CWP) is a complex pain condition characterized by generalized musculoskeletal pain and often associated with other symptoms. An important clinical feature is widespread increased pain sensitivity such as lowered pain thresholds for mechanical stimuli (pressure pain thresholds [PPT]). There is a growing interest in investigating the activated neurobiological mechanisms in CWP, which includes fibromyalgia. In CWP, strong significant correlations have been found between muscle protein patterns and PPT. This explorative proteomic study investigates the multivariate correlation pattern between plasma proteins and PPT in CWP and in healthy controls (CON). In addition, this study analyses whether the important proteins for PPT differ between the 2 groups.

Using 2-dimensional gel electrophoresis, we analyzed the plasma proteome of the CWP (n = 15) and the CON (n = 23) and proteins were identified using mass spectrometry. For both the CWP and the CON, the associations between the identified proteins and PPT were analyzed using orthogonal partial least square in 2 steps.

Significant associations between certain plasma proteins and PPT existed both in CWP (*R*^2^ = 0.95; *P* = .006) and in CON (*R*^2^ = 0.89; *P* < .001). For both groups of subjects, we found several proteins involved in PPT that reflect different biological processes. The plasma proteins as well as the biological processes involved in PPT differed markedly between the 2 groups of subjects.

This study suggests that plasma protein patterns are associated with pain thresholds in CWP. Using the plasma proteome profile of CWP to study potential biomarker candidates could provide a snapshot of ongoing systemic mechanisms in CWP.

## Abstract 2

Supplemental Digital Content is available in the text

## 1 Introduction

Chronic widespread pain (CWP) is characterized by generalized musculoskeletal pain and often is associated with symptoms such as psychological distress, insomnia, fatigue, and cognitive difficulties.^[1–3]^ CWP prevalence is 5% to 10%, with a higher prevalence in women.^[4–7]^ Chronic pain, including CWP, affects not only patients but also their families and society, leading to significant suffering and high socioeconomic burden.^[7]^

CWP diagnosis is generally based on the first part of the criteria of the American College of Rheumatology (1990) definition for fibromyalgia (FM).^[3]^ Other definitions of CWP and FM have been presented^[8,9]^ even though the implementation of the revised criteria still is limited. Peripheral and central mechanisms are believed to contribute to the clinical picture in CWP.^[10–12]^ Recently, the International Association for the Study of Pain has defined nociplastic pain as a new pain mechanistic descriptor; FM is classified as a nociplastic pain condition.^[13]^ Nociplastic pain includes increased anatomical spread of pain on the body and increased pain sensitivity. Increased mechanical pain sensitivity is not a requirement for the diagnosis of CWP, but it is often found during clinical examination of these patients. Clinically, deep muscle tissue pain sensitivity is assessed using semi-objective methods such as tender point examination or pain thresholds for mechanical stimuli (pressure pain thresholds [PPT]). Several mechanisms could be responsible for widespread hyperalgesia in FM/CWP:

(1) a continuous nociceptive input;
(2) peripheral nociceptor sensitization;
(3) secondary hyperalgesia in the primary pain region; and/or
(4) a generalized state of hypersensitivity.^[14]^

Assessments and treatment evaluations in chronic pain conditions, including CWP/FM, are hampered by the lack of valid biomarkers for activated nociceptive mechanisms and pain. For example, interdisciplinary rehabilitation interventions are at best associated with low to moderate effect sizes.^[15]^ This might be explained by the fact that neurobiological mechanisms are not fully understood nor targeted in interventions. Fortunately, there is a growing interest in investigating activated neurobiological mechanisms in CWP/FM. Hence, potential biochemical biomarkers for CWP/FM are increasingly being reported as possible tools for analyzing protein patterns, cytokines/chemokines, lipids, and metabolites in blood, cerebrospinal fluid, muscles, and saliva.^[16–24]^ Some of these studies are hypothesis-driven investigations of a few molecules, and others have an exploratory approach using *omics* in combination with advanced statistical techniques such as multivariate data analysis (MVDA). The omics approach is gaining increasing support as the understanding that pain involves a myriad of molecular changes is evolving.^[25]^ The proteome (ie, the protein composition of a tissue) is considered to be an ideal source of molecular information for monitoring changes in the phenotype^[25]^ because the proteome is constantly modulated by genome-environment interactions whereas the genome remains relatively stable.^[26]^ Plasma is suitable for proteomic analysis as it is easily accessible, has a rich protein content, and reflects ongoing peripheral responses in blood and in other tissues.^[27]^

We have previously reported significant differences in muscle protein patterns between CWP and controls^[28]^ and statistically significant correlations between muscle protein patterns, pain intensity, and PPT in the CWP cohort.^[29]^ In the same CWP cohorts and controls, significant group differences were observed in the plasma protein pattern^[16]^; 22 altered proteins were found in the same CWP cohort. Plasma cytokines and chemokines (ie, small molecules typically at picomolar levels) multivarietly correlate with PPT in CWP, but not in controls.^[23]^

To the best of our knowledge, no study has analyzed whether correlations exist between the plasma proteome (reflecting molecules typically at nano and micro molar levels) and PPT. Such information could improve the understanding of the mechanisms responsible for pain sensitivity. Therefore, this explorative proteomic pilot study investigates the multivariate correlation pattern between PPT and plasma proteins in CWP patients (typically with lowered PPT)^[30]^ and in controls. Within this aim, we analyzed whether the proteins involved in PPT differed between the 2 groups.

## 2 Material and methods

### 2.1 Subjects

The recruitment process and the inclusion and exclusion criteria for the patients with CWP and healthy controls (CON) have been previously described in detail.^[30]^ The following exclusion criteria were used: the use of anticoagulatory, opioid, or steroidal medication and a medical history record of bursitis, tendonitis, capsulitis, postoperative conditions in the neck/shoulder area, previous neck trauma, disorder of the spine, neurological disease, rheumatoid arthritis or any other systemic diseases, metabolic disease, malignancy, severe psychiatric illness, or pregnancy. In addition, patients who had difficulties understanding the Swedish were excluded from the study.

Women with CWP were recruited if they were former patients with CWP at the Pain and Rehabilitation Centre of the University Hospital, Linköping, Sweden or were members of an organization for FM patients. The healthy group (CON) consisted of women who were recruited through local newspaper advertisements. As reported in earlier studies, a total of 19 CWP and 24 CON were initially recruited in the original study.^[30–32]^ However, difficulties with blood sampling from 2 of the CWP subjects and difficulties with 2 plasma samples (1 CWP and 1 CON) were not sufficient for further proteomic analysis, which resulted in blood samples from 16 CWP and 23 CON.^[16]^ In addition, 1 of the CWP patients was excluded in the present study due to incomplete self-reported data and unclear diagnosis after detailed analysis. Hence, proteomic analyses were performed on plasma from 15 CWP patients and 23 CON.

To confirm eligibility, all participants received a standardized clinical examination. The American College of Rheumatology 90 criteria was used to classify CWP/FM.^[3]^ The examination was followed by a health questionnaire (see below). At the clinical examination, weight (kg) and height (m) were registered and body mass index (kg/m^2^) was calculated (weight/(height)^2^); these data have been reported elsewhere for the 2 groups and here are only given as background data.^[33]^

All research subjects signed a written consent before the start of the study but after receiving verbal and written information about the objectives and procedures of the study. The study was approved by the Regional Ethical Review Board in Linköping, Sweden (Dnr: M10-08, M233-09, Dnr: 2010/164-32). The study was performed according to the guidelines of the Declaration of Helsinki.

### 2.2 Methods

All subjects answered a health questionnaire, which consisted of the following items and scales.

#### 2.2.1 Demographic data

Each subject's birth year was registered, and age (years) was calculated.

#### 2.2.2 Pain intensity and psychological distress

We have recently reported levels of pain intensity and psychological distress for the present 2 groups of subjects^[33]^; this information is reported here only for background purposes (for details, see^[33]^). To register pain intensity in the whole body, we used an 11-grade (0–10) numeric rating scale: 0 indicating no pain at all and 10 indicating worst possible pain.^[34]^ To capture psychological distress, we used the hospital anxiety and depression scale (HADS), which measures anxiety and depression.^[35]^ HADS comprises 7 items for each scale: HAD-D – depression and HAD-A – anxiety. The subscale scores range between 0 and 21, with the lower score indicating the least depression and anxiety possible.^[35]^ A total HADS score (HADS-total), which combines both the anxiety and depression scores, was used to determine psychological distress. The HADS-total is based on a very large recent psychometric analysis of HADS in chronic pain patients.^[36]^

#### 2.2.3 PPT

As a part of the clinical examination, PPTs were determined using an electronic pressure algometer (Somedic, Hörby, Sweden); the methodology is described in detail elsewhere.^[29]^ The diameter of the contact area was 10 mm, and the pressure was applied perpendicularly to the skin at a speed of 30 kPa/s. The participants were instructed to mark their pain threshold by pressing a button as the sensation of pressure changed to pain. To determine the PPTs, the algometry was performed bilaterally over the medial, middle, and lateral part of the descending part of the trapezius muscle. All measurements were conducted twice in approximately 5-minute intervals. The PPT values were calculated as the mean of these two measurements of lateral, middle, and medial site on the right and left trapezius muscle. In the regression analyses, the mean values of the right and left trapezius measurements were used. Before the actual testing, the participants were given instructions and allowed to examine the testing procedure. Note that the PPTs, although not for the same number of subjects, are presented elsewhere.^[30]^

#### 2.2.4 Sample collection

All participants were asked not to take any nonsteroidal anti-inflammatory drugs for 7 days and/or paracetamol medication for 12 hours before the sampling. Venous blood samples were collected in Ethylenediaminetetraacetic acid (EDTA) vacutainer and centrifuged at 1000 × g for 15 minutes. Plasma samples were collected, aliquoted, and stored at −86°C until use. All samples were blinded before analysis.

#### 2.2.5 Proteomics – 2-dimensional gel electrophoresis (2-DE)

Here, we briefly summarise the 2-DE procedure as the procedure has been described in detail earlier.^[16,28,37]^ Depleted plasma samples containing 100 μg total protein were run in the first dimension. This was followed by second dimension separation using Ettan DALTsix Electrophoresis Unit (Amersham, Pharmacia, Uppsala, Sweden). The protein gels were fluorescently stained with SYPRO Ruby (Bio-Rad Laboratories, Hercules, CA). We visualized the stained protein pattern using a charge coupled device camera (VersaDoc Imaging system 4000 MP, Bio-Rad) and further analyzed and quantified the protein pattern using PDQuest Advanced (v. 8.0.1, Bio-Rad). The amount of protein in a certain spot was assessed as background corrected optical density, integrated over all pixels in the spot and expressed as integrated optical density. The parts per million values for all proteins were generated and were evaluated for differences between the groups using MVDA. Two preparative gels (1 pool from CWP and 1 from CON, containing 400 μg of total protein) for protein identifications were run according to the above described protocol.

#### 2.2.6 Protein identification

To identify proteins, spots of interest were excised from the gel, de-stained, tryptically digested, and prepared.^[28]^ Two mass spectrometry (MS) instruments were used for protein identification: ultrafleXtreme matrix-assisted laser desorption/ionization–time of flight (Bruker Daltonik GmbH, Bremen, Germany) and nano liquid chromatography system (EASY-nLC, Thermo Scientific, Waltham, MA) with a C18 column (100 mm × 0.75 μm, Agilent Technologies, Santa Clara, CA) coupled a LTQ Orbitrap Velos Pro mass spectrometer (Thermo Scientific).

#### 2.2.7 Database search

The strategies for database search have been described earlier.^[16,20]^ Hence, the acquired MS data from matrix-assisted laser desorption/ionization–time of flight analysis were pre-processed using flexAnalysis v. 3.4 (Bruker Daltonik), and the major peak list from each processed spectra was imported into the search engine ProteinProspector MS-Fit (v. 5.14.4), including the Swiss-Prot database v. 2015.3.5.^[16,20]^ The acquired MS data from the Orbitrap was analyzed with MaxQuant v. 1.5.8.3 (Max Planck Institute of Biochemistry, Martinsried, Germany) using the human UniProt/Swiss-Prot database (downloaded April 04, 2017) as described previously.^[20]^ The identified proteins were primarily divided into groups based on the UniProt database (www.uniprot.org) definition for biological processes.

### 2.3 Statistics

#### 2.3.1 Univariate statistics

For comparison of group values of background variables and PPT, Student *t* test and the non-parametric Mann–Whitney *U* test were applied using IBM SPSS v. 24.0 (version 24.0; IBM Corporation, Route 100 Somers, New York) for normal distributed data and for non-normal distributed data, respectively; *P* < .05 was considered significant in all analyses.

#### 2.3.2 MVDA

To investigating the multivariate correlations between the proteins (*X*-variables) and the pain thresholds (*Y*-variable), Orthogonal partial least squares (OPLS) regression analysis was applied using SIMCA-P+ (version 15.0; Sartorius Stedim Biotech, Umeå, Sweden).^[38]^ We followed the recommendations concerning MVDA for omics data.^[39]^ As this procedure has been described in detail elsewhere,^[16,20,28]^ we provide only a brief description.

Principal component analysis using SIMCA-P+ was used to check for multivariate outliers; no multivariate outliers were identified. OPLS was used for the regression analyses of PPT of the trapezius as *Y*-variable and the proteins as regressors (*X*-variables). Variables with variable influence on projection value (VIP) >1.0 (combined with jack-knifed 95% confidence intervals in the regression coefficients plot not including 0) and with absolute *p* (corr) ≥0.40 were considered significant; *p* (corr) is the loading of each variable scaled as a correlation coefficient and thus standardizing the range from −1 to +1.^[39]^ The OPLS analysis was made in 2 steps. In the first step, all proteins were included in the analysis. In the second step, the proteins with VIP ≥1.0 and *p* (corr) ≥0.40 were used in a new OPLS regression. This article presents the results from the second step. For each OPLS model, *R*^2^ describes the goodness of fit and *Q*^2^ describes goodness of prediction.^[38]^ Cross validated analysis of variance (CV-ANOVA) with a *P* ≤ .05 was used to validate the obtained model. SIMCA-P+ uses the non-linear iterative partial least squares algorithm to handle missing data: max 50% missing data for variables/scales and max 50% missing data for subjects.

### 2.4 Bioinformatics

Using the online database tool Search Tool for Retrieval of Interacting Genes/Proteins (STRING; version 11), we analyzed the protein-protein association network for the important proteins in CWP and CON separately. Protein accession numbers (UniProt) for the significant proteins (ie, proteins with VIP ≥1.0 and absolute *p* (corr) ≥0.40) from the final OPLS regression was entered in the search engine (multiple proteins) with the following parameters: organism was *Homo sapiens*, maximum number of interactions was query proteins only, interaction score was set to minimum required interaction score of high confidence (0.700), and a false discovery rate (FDR) ≤0.05 was used when classifying the Biological Process (Gene ontology; GO) of each protein. For each obtained network, PPI enrichment *P*-value and average local clustering coefficient were reported. In the network figures, each protein is represented by a colored node, and protein-protein interaction and association are represented by an edge visualized as a line. Higher combined confidence scores are represented by thicker lines/edges.

## 3 Results

### 3.1 Background data

Clinical background data for the 2 groups have been presented elsewhere as mentioned above and are summarized in Table 1.^[33]^ Hence, patients with CWP had significantly higher pain intensity, were significantly older, and reported significantly higher HADS-total compared to the CON (Table 1). No significant group differences were found for height, weight, and body mass index.

**Table 1:**
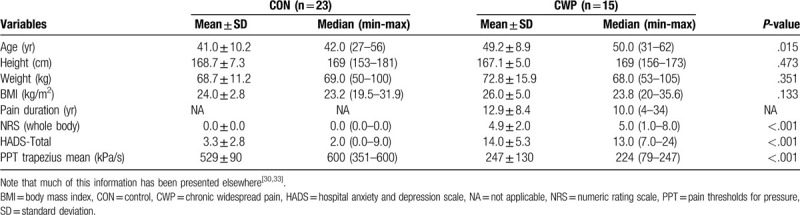
Demographic data, pain intensity (NRS) and other pain characteristics, psychological distress together with pain thresholds for pressure (PPT) of the trapezius presented as mean values (± 1 SD) and median (min-max).

### 3.2 PPTs

A significant difference in PPTs of the trapezius was found between the 2 groups of subjects (Table 1); these data have been presented earlier.^[30]^ Hence, as expected the CWP group displayed significantly lowered PPT.

### 3.3 2-DE analysis

As reported in the first proteomic study of these subjects, 414 ± 21 (CWP: 425 ± 18, CON: 408 ± 20) plasma proteins, including different isoforms (labeled as proteoforms below) from each gel, were detected in the 2-DE analysis,^[16]^ and 325 proteins were further quantified, matched, and analyzed using OPLS of PPT in the 2 groups of subjects. Another study of these subjects has reported the plasma protein pattern in relation to pain intensity and psychological distress.^[33]^

### 3.4 OPLS regressions of pain thresholds

Two OPLS models (1 for CWP and 1 for CON) were created to analyze the multivariate correlation pattern between identified plasma proteins and PPT in CWP and CON. According to the UniProt characterizations, the protein distribution in all models for the CWP and the CON groups were metabolic (30% and 25%, respectively), immunity (22% and 37%, respectively), iron ion homeostasis (26% and 19%, respectively), inflammatory (9% and 12%, respectively), and lipid metabolism (13% and 6%, respectively).

### 3.5 Plasma proteins as regressors of PPT in CWP

**Figure 1:**
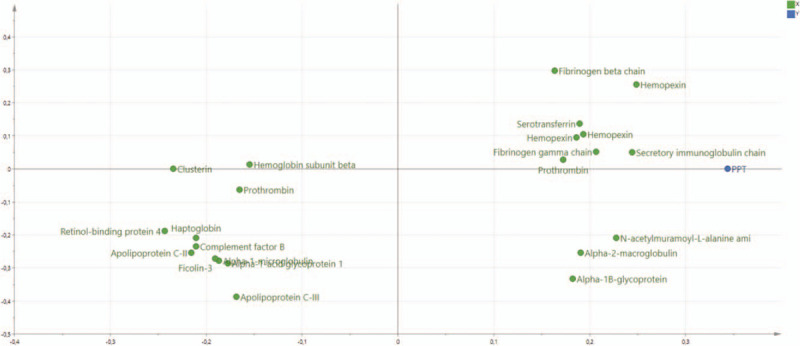
Orthogonal partial least squares regression analysis (OPLS) of PPT in CWP. Loading plots showing proteins with VIP ≥1.0 significantly correlated to PPT. CWP = chronic widespread pain, PPT = protein-protein interaction, VIP = variable influence on projection.

The final OPLS regression of PPT in CWP consisted of 1 predictive and 1 orthogonal component with a high goodness of fit (*R*^2^ = 0.95), goodness of prediction (*Q*^2^ = 0.74), and a significant CV-ANOVA (*P*-value = .006). A total of 23 proteins, including proteoforms, had VIP ≥1.0 and absolute *p* (corr) ≥0.40 (2 unidentified proteins were excluded) and were significant regressors of PPT in CWP (Table 2 and Fig. 1). According to the UniProt classification, the statistically significant proteins belonged to metabolic (n = 7 proteins), iron ion homeostasis (n = 6 proteins) and immunity processes (n = 5 proteins), and less significant proteins belonged to lipid metabolism (n = 3 proteins) and inflammatory processes (n = 2 proteins) (Table 2 and Fig. 1). The proteins with the strongest associations with PPT (ie, with VIP ≥1.4) were hemopexin (2 proteoforms; iron ion homeostasis according to UniProt), retinol-binding protein 4 (metabolic), secretory immunoglobulin chain α (immunity), apolipoprotein C-II (lipid metabolism), complement factor B (Immunity), and clusterin (lipid metabolism).

**Table 2:**
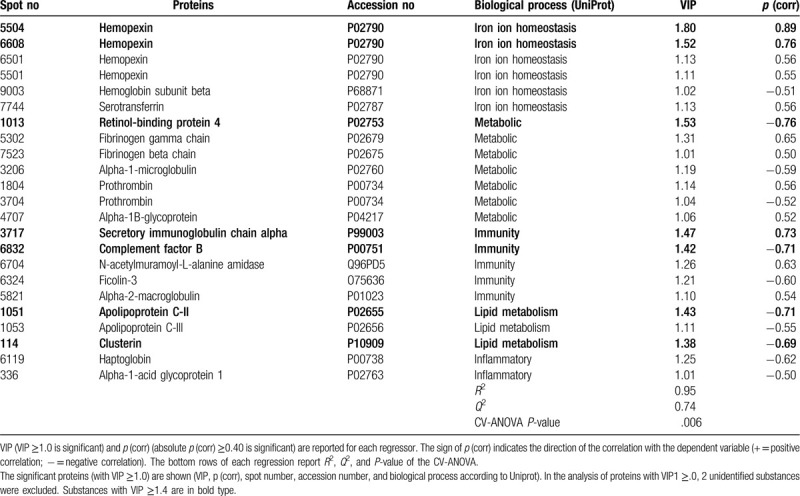
Orthogonal partial least squares regression (OPLS) of PPT in CWP.

### 3.6 Plasma proteins as regressors of PPT in CON

**Figure 2:**
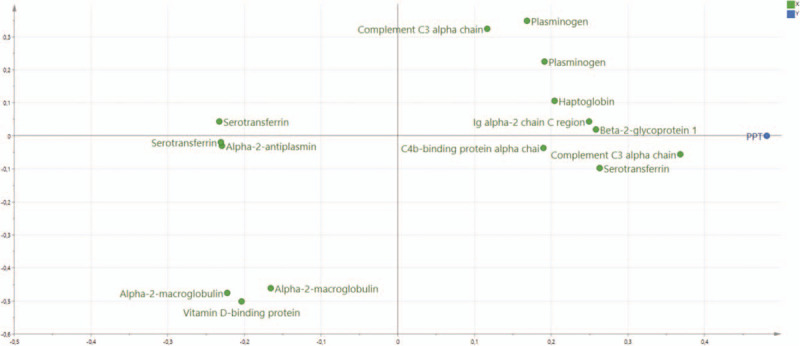
Orthogonal partial least squares regression analysis (OPLS) of PPT in CON. Loading plots showing proteins with VIP ≥1.0 significantly correlated to PPT. CON = healthy controls, PPT = protein-protein interaction, VIP = variable influence on projection.

The final OPLS analysis of PPT in CON consisted of 1 predictive and 1 orthogonal component with a high goodness of fit (*R*^2^ = 0.89), goodness of prediction (*Q*^2^ = 0.76), and a significant CV-ANOVA (*P*-value < .001). Sixteen proteins, including proteoforms, had VIP ≥1.0 and absolute *p* (corr) ≥0.40 and were considered as significant regressors for PPT in CON (Table 3 and Fig. 2). According to the UniProt classification, the majority of the significant proteins belonged to immunity processes (n = 6 proteins), metabolic (n = 4 proteins), iron ion homeostasis (n = 3 proteins), whereas less significant proteins belonged to inflammatory processes (n = 2 proteins) and lipid metabolism (n = 1 protein) (Table 3 and Fig. 2). The proteins with the highest VIP values (≥1.40) and; therefore, the strongest associations with PPT in CON were 3 proteins associated with immunity according to UniProt: complement C3 alpha chain, Ig alpha-2 chain C region, and alpha-2-macroglobulin. In addition, vitamin D-binding protein was associated with inflammation.

**Table 3:**
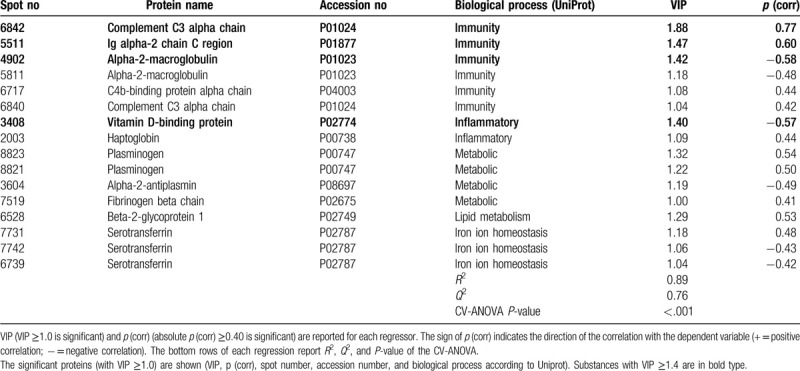
Orthogonal partial least squares regression (OPLS) of PPT in CON.

### 3.7 Pathway analyses

#### 3.7.1 CWP

**Figure 3:**
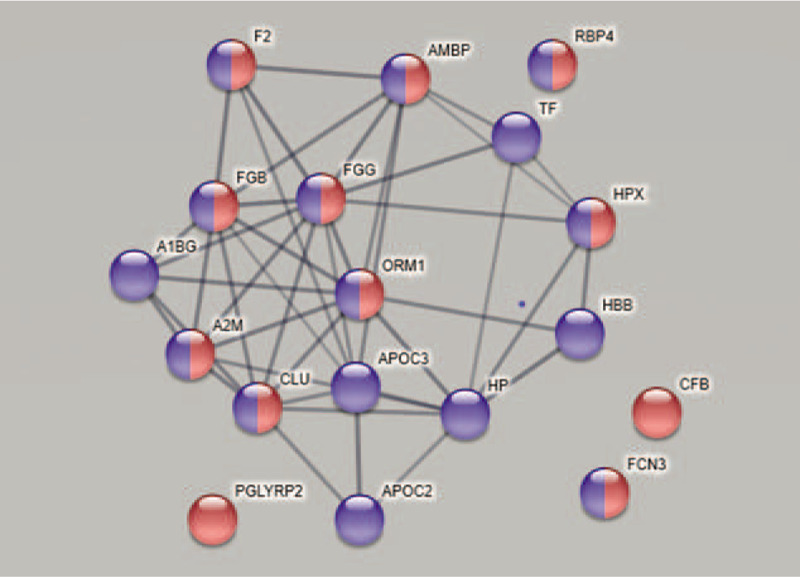
Network and enrichment analysis of 19 significantly correlated proteins with PPT in CWP based on STRING database using a high confidence (minimum required interaction score 0.700). Note that STRING did not include secretory immunoglobulin chain alpha (P99003). The confidence score represents the probability that the marked interaction is biologically meaningful based on the supporting evidence (ref). Higher combined confidence scores are represented by thicker lines. The observed protein-protein interactions were significantly enriched (PPI enrichment *P*-value < 1.0e-16) and with average local clustering coefficient of 0.514, indicating that these differentially expressed proteins are biologically connected. The proteins were related to immune response with FDR 6.12e-08 (highlighted red) and to transport with FDR 2.95e-07 (highlighted in blue). The nodes are marked with the gene name of the proteins and the corresponding protein name: A1BG = alpha-1B-glycoprotein, A2M = alpha-2-macroglobulin, AMBP = alpha-1-microglobulin, APOC2 = apolipoprotein C-II, APOC3 = apolipoprotein C-III, CFB = complement factor B, CLU = clusterin, F2 = prothrombin, FCN3 = ficolin-3, FDR = false discovery rate, FGB = fibrinogen beta chain, FGG = fibrinogen gamma chain, HBB = hemoglobin subunit beta, HP = haptoglobin, HPX = hemopexin, ORM1 = alpha-1-acid glycoprotein 1, PGLYRP2 = N-acetylmuramoyl-L-alanine amidase, PPI = protein-protein interaction, PPT = pressure pain threshold, RBP4 = retinol-binding protein 4, STRING = search tool for retrieval of interacting genes/proteins, TF = serotransferrin.

The network and enrichment analysis of the 17 significantly correlated proteins with PPT in CWP (Table 2) based on STRING database identified a protein-protein interaction network that was significantly enriched (PPI enrichment *P*-value < 1.0e-16) (Fig. 3). However, it was not possible to include proteoforms and STRING did not include Secretory immunoglobulin chain alpha (P99003). The proteins were related to immune response (FDR = 6.12e-08) and to transport (FDR = 2.95e-07). Hence, according to the network analysis, most of the identified proteins in the OPLS are biologically connected (Fig. 3). Four proteins did not show significant interactions with the rest of the proteins: Retinol-binding protein 4, Complement factor B, Ficolin-3, and N-acetylmuramoyl-L-alanine amidase (PGLYRP2). However, the network also included the first 3 of these 4 proteins when using a lower minimum required interaction score (ie, medium confidence 0.400).

#### 3.7.2 CON

**Figure 4:**
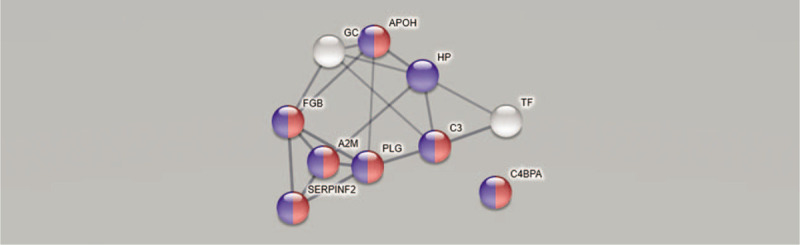
Network and enrichment analysis of 10 significantly correlated proteins with PPT in healthy subjects (CON) based on STRING database using a high confidence (minimum required interaction score 0.700). Note that STRING did not include Ig alpha-2 chain C region (P01877). The confidence score represents the probability that the marked interaction is biologically meaningful based on the supporting evidence. Higher combined confidence scores are represented by thicker lines. The observed protein-protein interactions were significantly enriched (PPI enrichment *P*-value < 1.0e-16) and with an average clustering coefficient of 0.503 indicating that these differentially expressed proteins are biologically connected. Majority of the proteins were related to regulation of response to external stimuli with FDR 8.65e-07 (highlighted red,) and response to stress FDR 1.42e-05 (highlighted in blue). The nodes are marked with the gene name of the proteins and the corresponding protein name were: A2M = alpha-2-macroglobulin, APOH = beta-2-glycoprotein 1, C3 = complement C3 alpha chain, C4BPA = C4b-binding protein alpha chain, FDR = false discovery rate, FGB = fibrinogen beta chain, GC = vitamin D-binding protein, HP = haptoglobin, PLG = plasminogen, PPI = protein-protein interaction, PPT = pressure pain threshold, SERPINF2 = alpha-2-antiplasmin, STRING = search tool for retrieval of interacting genes/proteins, TF = serotransferrin.

The network and enrichment analysis of 10 significantly correlated proteins with PPT in CON (Table 3) based on STRING database identified a protein-protein interaction network that was significantly enriched (PPI enrichment *P*-value < 1.0e-16) (Fig. 4). However, it was not possible to include proteoforms and STRING did not include Ig alpha-2 chain C region (P01877). This analysis indicated that these differentially expressed proteins are biologically connected. Most of the proteins were related to the regulation of response to external stimuli (FDR = 8.65e-07) and to response to stress (FDR = 1.42e-05). C4b-binding protein alpha chain did not show significant interactions with the rest of the proteins in the network. However, the network also included this protein when using a lower minimum required interaction score (ie, medium confidence 0.400).

## 4 Discussion

### 4.1 Major results

This study has 3 important and novel results:

- Significant and strong associations between certain plasma proteins and PPT existed both in CWP and in CON.
- The important plasma proteins and the involved biological processes for PPT differed markedly between the 2 groups of subjects.
- Both groups of subjects had multiple proteins involved in PPT and these proteins formed highly enriched networks

### 4.2 Peripheral factors associated with PPT

Knowledge about molecular mechanisms underlying increased pain sensitivity (eg, lowered PPT) is lacking. Moreover, explorative investigations of the plasma proteome in CWP/FM have been limited. To date, no studies have investigated the relationship between PPT and the plasma proteomic profile. Typically, proteomics can be used to analyze proteins at nano and micro molar concentrations while cytokines are found at picomolar concentrations. In the same CWP cohort, we reported that 6 plasma cytokines and chemokines (4E-BP1, Hepatocyte growth factor, CASP8, MIP1-α, CDCP1, and STAMPB) were significantly associated with PPT.^[23]^ However, this regression was weaker (*R*^2^ = 0.44, *Q*^2^ = 0.34, CV-ANOVA *P*-value = .011) than the present regression in CWP (*R*^2^ = 0.95, *Q*^2^ = 0.74, CV-ANOVA *P*-value = .006) (Table 2). Hence, the proteins captured via the proteomic profile explained much more of the variation in PPT than cytokines/chemokines. Moreover, from the same CWP cohort, we investigated the proteome of muscle biopsies from the trapezius muscle and found significant associations between certain proteins and PPT (*R*^2^ = 0.95, *Q*^2^ = 0.81, CV-ANOVA *P*-value < .05).^[29]^ Taken together, these studies indicate that proteins in muscle and blood are significantly correlated with mechanical pain sensitivity in the CWP (mainly FM) and in the CON.

### 4.3 The biological processes in the 2 groups

As evident from the two regressions, the proteins involved in PPT were very different in the two groups (Tables 2 and 3). These proteins (VIP ≥1.4) were not at all identical in the 2 groups (Tables 2 and 3). According to the UniProt classification 3 immunity proteins were strongest associated with PPT in the CON, whereas the most involved proteins for PPT in CWP reflected different biological processes. Although Figures 1 and 2 show that several of the proteins that explain the variations in PPT in the two groups in fact are intercorrelated, it is also important to investigate whether there exists bioinformatic support that these proteins form protein-protein interaction networks. Indeed, we found that most of the important proteins intercorrelated in the multivariate context (Figs. 1 and 2) and that there was bioinformatic support for these associations (Figs. 3 and 4). Hence, the protein-protein interaction analyses resulting in significantly enriched networks clearly indicate that different biological processes are important for explaining variations in PPT in the 2 groups. Hence, in the CON, the significant and interacting proteins reflect responses to external stimuli and stress. In the CWP; however, the protein interaction network is related to immune response and to transportation. Hence, differences in PPT between the 2 groups are not a scaling issue for the same proteins. Rather, the lowered PPT in the CWP is associated with mechanisms other than what is found in the CON. Furthermore, the identified networks in turn indicate that more complex relationships exist and that no single protein, at least as far as we know, can explain PPT.

### 4.4 Why is the protein pattern in plasma important for PPT?

PPT measures when an acute mechanical stimulus becomes painful; in healthy subjects, the stimuli both activates peripheral nociceptors and the CNS. The levels of certain proteins in plasma from the CON were associated with the report that the stimulus was painful. In the CON, the network analysis showed that the involved proteins were important in biological processes such as responses to stress and to external stimuli. The levels of these significant proteins were highly associated with PPT in the CON. It is noteworthy that it was not possible to significantly regress PPT in the CON using cytokines/chemokines.^[23]^ Our study found that other non-inflammatory protein networks are important for PPT in the CON. The strong significant associations (*R*^2^ = 0.89) might indicate that primary mechanisms involved in the PPT response have been captured, but this is a cross-sectional cohort study so causal relationships cannot be disentangled.

CWP/FM is associated with morphological and functional changes in the brain, neuroinflammation, opioidergic dysregulation, and central sensitization (ie, nociception-driven amplification of neural signaling and/or impaired top-down modulation) as well as peripheral alterations such as systemic low-grade inflammation and alterations in nociceptor and muscle.^[40–49]^ These alterations indicate complicated interactions between peripheral and central processes in CWP/FM.

Generalized hyperalgesia (ie, lowered pain thresholds) is a criteria of FM and generally, as in the present study, are found in CWP.^[30,50]^ This hyperalgesia is clinically often attributed to central sensitization, including impaired descending control of nociception, and is associated with activation of the central nervous system (CNS) network composed of the anterior cingulate, insula, and prefrontal cortex.^[51]^ These and other central alterations in the CNS might be independent of or dependent on peripheral input. The acute stimuli associated with the PPT registration is a peripheral input, but there might also exist a more continuous nociceptive input to the CNS. It has been suggested that different types of peripheral stimuli lead to long-term potentiation and “wind up.”^[52]^ Goubert et al suggest that peripheral injury/stressors trigger pro-inflammatory cytokines contributing to central sensitization.^[53]^ The identified plasma proteins in CWP might reflect an ongoing abnormal peripheral input into the CNS, including effects of antidromic firing, which taken together contribute to the initiation and maintenance of the hyperalgesic state.^[12,54,55]^ It is possible that in CWP maintained hyperalgesic priming of peripheral nociceptors and peripheral nociceptor sensitization, clinically manifested as hyperalgesia in the primary pain area, is maintained or increased by the presence of abnormal peripheral input. In addition, maintained peripheral input is reasonably associated with secondary hyperalgesia.

Ji et al conclude that central sensitization can be driven by neuroinflammation in the peripheral and CNS and that the central sensitization drives CWP.^[56]^ Information on peripheral inflammatory activity is transmitted via peripheral nociceptors, humoral, and neuronal (eg, the vagus nerve) pathways to the CNS, resulting in, for example, sickness behavior, decreased endogenous pain inhibition, and neuroinflammation.^[57–61]^ There is emerging evidence that both FM and CWP are associated with neuroinflammation in the CNS.^[20,62]^ Neuro-inflammation in the CNS is associated with increased blood-brain barrier permeability,^[56]^ and pro-inflammatory cytokines can cross the blood-brain barrier and enter the CNS.^[63]^ The inflammatory cytokine/chemokine profile in plasma suggested low-grade inflammation in the present CWP cohort and in a FM cohort.^[23,62]^ A recent review highlighted immunological factors and broadened the perspective beyond cytokines/chemokines, suggesting that lipid mediators, oxidative stress, and several plasma-derived factors might also contribute to an inflammatory state in FM.^[47]^ Hence, there are reports that CWP/FM have altered plasma levels of glutamate, lactate, nerve growth factor, brain-derived neurotrophic factor, and lipid mediators.^[24,30,50,64]^ Moreover, we recently reported prominent differences in the plasma proteome between CWP and CON.^[16]^ In the present patients, plasma cytokines/chemokines showed significant associations with PPT, but the associations presented in this plasma proteomic study are markedly stronger (see above). This finding suggests that a broader perspective than only focusing on certain cytokines/chemokines is necessary when trying to capture the group of molecules associated with PPT.

CNS also affects peripheral immune activation.^[65,66]^ Bidirectional signaling pathways exist between the immune and nervous systems both at peripheral and central levels.^[58,61,65,67–69]^ The CNS exerts top-down effects on peripheral inflammatory activity via neuroendocrine and autonomic effector mechanisms.^[65]^ The neuroendocrine mechanisms involve the hypothalamic-pituitary-adrenal axis and result in peripheral modulations by glucocorticoids as the result of inflammatory mechanisms.^[65]^ For example, dysregulation of hypothalamic-pituitary-adrenal axis has been found in FM.^[70]^ Peripheral proinflammatory actions via the sympathetic nervous system and down-regulating peripheral inflammatory activity via the parasympathetic nervous system are possible actions of the autonomic system.^[65]^

To summarize, there are several possible mechanisms – not mutually exclusive – involved in widespread hyperalgesia: a more or less continuous nociceptive input, peripheral nociceptor sensitization, secondary hyperalgesia in the primary pain region, and a generalized state of hypersensitivity.^[14]^ The identified proteins might correlate with 1 or several of these mechanisms.

### 4.5 The most important proteins for PPT

Although the proteins involved in networks reflect different biological processes in the 2 groups, these proteins might also provide valuable knowledge about the role of the most important proteins (VIP ≥1.4) for regressing PPT in both groups, including their relation to pain aspects in CWP. This information is summarized in the digital supplemental text, http://links.lww.com/MD/E292.

### 4.6 Strengths and limitations

Obvious limitations are the small sample size and the cross-sectional design. To validate these results, larger studies with repeated measures of PPT and blood samples are needed. Moreover, it is important to investigate if significant associations exist between the plasma protein pattern and PPT in other chronic pain diagnoses and if so to what extent the important proteins are identical with those found in CWP. MVDA have been increasingly applied in proteomic studies of tissues from chronic pain patients^[16,18,62,71,72]^; our studies demonstrate that MVDA is highly applicable both for differentiating patients from controls and for relating clinical parameters such as PPT to plasma proteins. The necessary removal procedure of large abundant proteins (ie, albumin and IgG) could have removed other and low abundant proteins that could be of interest. An advantage of 2-DE is its ability to detect different proteoforms of a protein and post translational modifications. In this study and our recent studies, this is apparently of importance since several of the significant proteins were expressed as different proteoforms.^[16,33]^ The relevancies of post translational modifications in the chronic pain context are not understood and further research is warranted.

## 5 Conclusions

Plasma protein patterns are associated with PPT in the CWP and the CON. In both regressions, a panel of representative proteins were found to be involved in PPT. However, these proteins and the biological process were different between the two groups. Insights into ongoing systemic mechanisms in CWP could be gained by studying the plasma proteome profile of CWP and potential biomarker candidates for lowered PPT (ie, increased pain sensitivity). From a systemic perspective, the effect might be local peripheral changes in muscles and plasma and/or central changes such as central sensitization. Larger studies, including longitudinal studies, that capture more proteins are needed to validate our results and increase knowledge about the molecular events that increase pain sensitivity at the peripheral and systemic levels.

## Acknowledgment

The authors would like to thank research nurse Eva-Britt Lind at Pain and Rehabilitation Centre, Region Östergötland, Linköping, for the valuable help during the recruitment process and sample collection.

## Author contributions

BGh and BGe contributed to the conception of the study. KW performed the experiments and analyzed all the data. KW and BGe drafted the manuscript. All authors contributed to the writing of the manuscript and approved the final version.

## Supplementary Material

SUPPLEMENTARY MATERIAL

## References

[1] Perez de Heredia-Torres M Huertas-Hoyas E Maximo-Bocanegra N. Cognitive performance in women with fibromyalgia: a case-control study. Aust Occup Ther J 2016;63:329–37. 27059423 10.1111/1440-1630.12292

[2] Aparicio VA Ortega FB Carbonell-Baeza A. Fibromyalgia's key symptoms in normal-weight, overweight, and obese female patients. Pain Manag Nurs 2013;14:268–76. 24315250 10.1016/j.pmn.2011.06.002

[3] Wolfe F Smythe HA Yunus MB. The American College of Rheumatology 1990 criteria for the classification of fibromyalgia. Report of the multicenter criteria committee. Arthritis Rheum 1990;33:160–72. 2306288 10.1002/art.1780330203

[4] Mansfield KE Sim J Jordan JL. A systematic review and meta-analysis of the prevalence of chronic widespread pain in the general population. Pain 2016;157:55–64. 26270591 10.1097/j.pain.0000000000000314 PMC4711387

[5] Cimmino MA Ferrone C Cutolo M. Epidemiology of chronic musculoskeletal pain. Best Pract Res Clin Rheumatol 2011;25:173–83. 22094194 10.1016/j.berh.2010.01.012

[6] Bergman S Herrstrom P Hogstrom K. Chronic musculoskeletal pain, prevalence rates, and sociodemographic associations in a Swedish population study. J Rheumatol 2001;28:1369–77. 11409133

[7] Breivik H Collett B Ventafridda V. Survey of chronic pain in Europe: prevalence, impact on daily life, and treatment. Eur J Pain 2006;10:287–333. 16095934 10.1016/j.ejpain.2005.06.009

[8] Arnold LM Bennett RM Crofford LJ. AAPT diagnostic criteria for fibromyalgia. J Pain 2019;20:611–28. 30453109 10.1016/j.jpain.2018.10.008

[9] Wolfe F Clauw DJ Fitzcharles MA. 2016 Revisions to the 2010/2011 fibromyalgia diagnostic criteria. Semin Arthritis Rheum 2016;46:319–29. 27916278 10.1016/j.semarthrit.2016.08.012

[10] Flodin P Martinsen S Lofgren M. Fibromyalgia is associated with decreased connectivity between pain- and sensorimotor brain areas. Brain Connect 2014;4:587–94. 24998297 10.1089/brain.2014.0274 PMC4202907

[11] Staud R Vierck CJ Cannon RL. Abnormal sensitization and temporal summation of second pain (wind-up) in patients with fibromyalgia syndrome. Pain 2001;91:165–75. 11240089 10.1016/s0304-3959(00)00432-2

[12] Staud R Nagel S Robinson ME. Enhanced central pain processing of fibromyalgia patients is maintained by muscle afferent input: a randomized, double-blind, placebo-controlled study. Pain 2009;145:96–104. 19540671 10.1016/j.pain.2009.05.020 PMC2751583

[13] Kosek E Cohen M Baron R. Do we need a third mechanistic descriptor for chronic pain states? Pain 2016;157:1382–6. 26835783 10.1097/j.pain.0000000000000507

[14] Sjors A Larsson B Persson AL. An increased response to experimental muscle pain is related to psychological status in women with chronic non-traumatic neck-shoulder pain. BMC Musculoskelet Disord 2011;12:230. 21992460 10.1186/1471-2474-12-230 PMC3204274

[15] Ringqvist Å Dragioti E Björk M. Moderate and stable pain reductions as a result of interdisciplinary pain rehabilitation – a cohort study from the Swedish Quality Registry for Pain Rehabilitation (SQRP). J Clin Med 2019;8:E905. 10.3390/jcm8060905 PMC6617026 31238588

[16] Wåhlén K Olausson P Carlsson A. Systemic alterations in plasma proteins from women with chronic widespread pain compared to healthy controls: a proteomic study. J Pain Res 2017;10:797–809. 28435317 10.2147/JPR.S128597 PMC5388344

[17] Olausson P Gerdle B Ghafouri N. Identification of proteins from interstitium of trapezius muscle in women with chronic myalgia using microdialysis in combination with proteomics. PloS One 2012;7:e52560. 23300707 10.1371/journal.pone.0052560 PMC3531451

[18] Hadrevi J Bjorklund M Kosek E. Systemic differences in serum metabolome: a cross sectional comparison of women with localised and widespread pain and controls. Sci Rep 2015;5:15925. 26522699 10.1038/srep15925 PMC4629114

[19] Culic O Cordero MD Zanic-Grubisic T. Serum activities of adenosine deaminase, dipeptidyl peptidase IV and prolyl endopeptidase in patients with fibromyalgia: diagnostic implications. Clin Rheumatol 2016;35:2565–71. 27527091 10.1007/s10067-016-3377-8

[20] Olausson P Ghafouri B Backryd E. Clear differences in cerebrospinal fluid proteome between women with chronic widespread pain and healthy women - a multivariate explorative cross-sectional study. J Pain Res 2017;10:575–90. 28331360 10.2147/JPR.S125667 PMC5356922

[21] Zanette SA Dussan-Sarria JA Souza A. Higher serum S100B and BDNF levels are correlated with a lower pressure-pain threshold in fibromyalgia. Mol Pain 2014;10:46. 25005881 10.1186/1744-8069-10-46 PMC4094546

[22] Bazzichi L Ciregia F Giusti L. Detection of potential markers of primary fibromyalgia syndrome in human saliva. Proteomics Clin Appl 2009;3:1296–304. 21136951 10.1002/prca.200900076

[23] Gerdle B Ghafouri B Ghafouri N. Signs of ongoing inflammation in female patients with chronic widespread pain: a multivariate, explorative, cross-sectional study of blood samples. Medicine (Baltimore) 2017;96:e6130. 28248866 10.1097/MD.0000000000006130 PMC5340439

[24] Jablochkova A Backryd E Kosek E. Unaltered low nerve growth factor and high brain-derived neurotrophic factor levels in plasma from patients with fibromyalgia after a 15-week progressive resistance exercise. J Rehabil Med 2019;51:779–87. 31544950 10.2340/16501977-2593

[25] Gomez-Varela D Barry AM Schmidt M. Proteome-based systems biology in chronic pain. J Proteomics 2019;190:1–1. 29653266 10.1016/j.jprot.2018.04.004

[26] Niederberger E Geisslinger G. Proteomics in neuropathic pain research. Anesthesiology 2008;108:314–23. 18212577 10.1097/01.anes.0000299838.13368.6e

[27] Chromy BA Gonzales AD Perkins J. Proteomic analysis of human serum by two-dimensional differential gel electrophoresis after depletion of high-abundant proteins. J Proteome Res 2004;3:1120–7. 15595720 10.1021/pr049921p

[28] Olausson P Gerdle B Ghafouri N. Protein alterations in women with chronic widespread pain--an explorative proteomic study of the trapezius muscle. Sci Rep 2015;5:11894. 26150212 10.1038/srep11894 PMC4493691

[29] Olausson P Ghafouri B Ghafouri N. Specific proteins of the trapezius muscle correlate with pain intensity and sensitivity - an explorative multivariate proteomic study of the trapezius muscle in women with chronic widespread pain. J Pain Res 2016;9:345–56. 27330327 10.2147/JPR.S102275 PMC4898258

[30] Gerdle B Larsson B Forsberg F. Chronic widespread pain: increased glutamate and lactate concentrations in the trapezius muscle and plasma. Clin J Pain 2014;30:409–20. 23887335 10.1097/AJP.0b013e31829e9d2a

[31] Gerdle B Soderberg K Salvador Puigvert L. Increased interstitial concentrations of pyruvate and lactate in the trapezius muscle of patients with fibromyalgia: a microdialysis study. J Rehabil Med 2010;42:679–87. 20603699 10.2340/16501977-0581

[32] Ghafouri N Ghafouri B Larsson B. Palmitoylethanolamide and stearoylethanolamide levels in the interstitium of the trapezius muscle of women with chronic widespread pain and chronic neck-shoulder pain correlate with pain intensity and sensitivity. Pain 2013;154:1649–58. 23707281 10.1016/j.pain.2013.05.002

[33] Wåhlén K Ghafouri B Ghafouri N. Plasma protein pattern correlates with pain intensity and psychological distress in women with chronic widespread pain. Front Psychol 2018;9:2400. 30555396 10.3389/fpsyg.2018.02400 PMC6281753

[34] Ferreira-Valente MA Pais-Ribeiro JL Jensen MP. Validity of four pain intensity rating scales. Pain 2011;152:2399–404. 21856077 10.1016/j.pain.2011.07.005

[35] Zigmond AS Snaith RP. The hospital anxiety and depression scale. Acta Psychiatrica Scandinavica 1983;67:361–70. 6880820 10.1111/j.1600-0447.1983.tb09716.x

[36] LoMartire R, Äng B, Gerdle B, Vixner L. Psychometric properties of SF-36, EQ-5D and HADS in patients with chronic pain. *Pain*. 161:83-95, 2020 10.1097/j.pain.0000000000001700 PMC6940032 31568237

[37] Gorg A Drews O Luck C. 2-DE with IPGs. Electrophoresis 2009;30: (Suppl 1): S122–32. 19441019 10.1002/elps.200900051

[38] Eriksson L Byrne T Johansson E. Multi- and Megavariate Data Analysis: Basic Principles and Applications. Third revised edition Malmö: MKS Umetrics AB; 2013.

[39] Wheelock AM Wheelock CE. Trials and tribulations of ’omics data analysis: assessing quality of SIMCA-based multivariate models using examples from pulmonary medicine. Mol Biosyst 2013;9:2589–96. 23999822 10.1039/c3mb70194h

[40] Sluka KA Clauw DJ. Neurobiology of fibromyalgia and chronic widespread pain. Neuroscience 2016;338:114–29. 27291641 10.1016/j.neuroscience.2016.06.006 PMC5083139

[41] Schrepf A Harper DE Harte SE. Endogenous opioidergic dysregulation of pain in fibromyalgia: a PET and fMRI study. Pain 2016;157:2217–25. 27420606 10.1097/j.pain.0000000000000633 PMC5028286

[42] Üçeyler N Sommer C. Häuser W Perrot S. Small nerve fiber pathology. Fibromylagia Syndrome and Widespread Pain - From Construction to Relevant Recognition. Philadelphia: Wolters Kluwer; 2018. 204–14.

[43] Gerdle B Larsson B. Häuser W Perrot S. Muscle. Fibromyalgia Syndrome and Widespread Pain – From Construction to Relevant Recognition. Philadelphia: Wolters Kluwer; 2018. 215–31.

[44] Albrecht DS Forsberg A Sandstrom A. Brain glial activation in fibromyalgia - a multi-site positron emission tomography investigation. Brain Behav Immun 2019;75:72–83. 30223011 10.1016/j.bbi.2018.09.018 PMC6541932

[45] Jensen KB Kosek E Petzke F. Evidence of dysfunctional pain inhibition in fibromyalgia reflected in rACC during provoked pain. Pain 2009;144:95–100. 19410366 10.1016/j.pain.2009.03.018

[46] Littlejohn G Guymer E. Neurogenic inflammation in fibromyalgia. Semin Immunopathol 2018;40:291–300. 29556959 10.1007/s00281-018-0672-2

[47] Coskun Benlidayi I. Role of inflammation in the pathogenesis and treatment of fibromyalgia. Rheumatol Int 2019;39:781–91. 30756137 10.1007/s00296-019-04251-6

[48] van Ettinger-Veenstra H Lundberg P Alfoldi P. Chronic widespread pain patients show disrupted cortical connectivity in default mode and salience networks, modulated by pain sensitivity. J Pain Res 2019;12:1743–55. 31213886 10.2147/JPR.S189443 PMC6549756

[49] Goubert D Danneels L Graven-Nielsen T. Differences in pain processing between patients with chronic low back pain, recurrent low back pain, and fibromyalgia. Pain Physician 2017;20:307–18. 28535553

[50] Stensson N Ghafouri N Ernberg M. The relationship of endocannabinoidome lipid mediators with pain and psychological stress in women with fibromyalgia: a case-control study. J Pain 2018;19:1318–28. 29885369 10.1016/j.jpain.2018.05.008

[51] Pujol J Macia D Garcia-Fontanals A. The contribution of sensory system functional connectivity reduction to clinical pain in fibromyalgia. Pain 2014;155:1492–503. 24792477 10.1016/j.pain.2014.04.028

[52] Eller-Smith OC Nicol AL Christianson JA. Potential mechanisms underlying centralized pain and emerging therapeutic interventions. Front Cell Neurosci 2018;12:35. 29487504 10.3389/fncel.2018.00035 PMC5816755

[53] Goubert D Meeus M Willems T. The association between back muscle characteristics and pressure pain sensitivity in low back pain patients. Scand J Pain 2018;18:281–93. 29794309 10.1515/sjpain-2017-0142

[54] Vierck CJJ. Mechanisms underlying development of spatially distributed chronic pain (fibromyalgia). Pain 2006;124:242–63. 16842915 10.1016/j.pain.2006.06.001

[55] Schneider GM Smith AD Hooper A. Minimizing the source of nociception and its concurrent effect on sensory hypersensitivity: an exploratory study in chronic whiplash patients. BMC Musculoskelet Disord 2010;11:29. 20144214 10.1186/1471-2474-11-29 PMC2829507

[56] Ji RR Nackley A Huh Y. Neuroinflammation and central sensitization in chronic and widespread pain. Anesthesiology 2018;129:343–66. 29462012 10.1097/ALN.0000000000002130 PMC6051899

[57] Karshikoff B Jensen KB Kosek E. Why sickness hurts: a central mechanism for pain induced by peripheral inflammation. Brain Behav Immun 2016;57:38–46. 27058164 10.1016/j.bbi.2016.04.001

[58] Eisenberger NI Moieni M Inagaki TK. In Sickness and in health: the co-regulation of inflammation and social behavior. Neuropsychopharmacology 2017;42:242–53. 27480575 10.1038/npp.2016.141 PMC5143485

[59] Lampa J Westman M Kadetoff D. Peripheral inflammatory disease associated with centrally activated IL-1 system in humans and mice. Proc Natl Acad Sci U S A 2012;109:12728–33. 22802629 10.1073/pnas.1118748109 PMC3411968

[60] Kempuraj D Thangavel R Selvakumar GP. Brain and peripheral atypical inflammatory mediators potentiate neuroinflammation and neurodegeneration. Front Cell Neurosci 2017;11:216. 28790893 10.3389/fncel.2017.00216 PMC5522882

[61] Karshikoff B Tadros M Mackey S. Neuroimmune modulation of pain across the developmental spectrum. Curr Opin Behav Sci 2019;28:85–92. 32190717 10.1016/j.cobeha.2019.01.010 PMC7079707

[62] Bäckryd E Tanum L Lind AL. Evidence of both systemic inflammation and neuroinflammation in fibromyalgia patients, as assessed by a multiplex protein panel applied to the cerebrospinal fluid and to plasma. J Pain Res 2017;10:515–25. 28424559 10.2147/JPR.S128508 PMC5344444

[63] Palada V Ahmed AS Finn A. Characterization of neuroinflammation and periphery-to-CNS inflammatory cross-talk in patients with disc herniation and degenerative disc disease. Brain Behav Immun 2019;75:60–71. 30248387 10.1016/j.bbi.2018.09.010

[64] Stensson N Ghafouri B Gerdle B. Alterations of anti-inflammatory lipids in plasma from women with chronic widespread pain - a case control study. Lipids Health Dis 2017;16:112. 28606089 10.1186/s12944-017-0505-7 PMC5469054

[65] Kraynak TE Marsland AL Wager TD. Functional neuroanatomy of peripheral inflammatory physiology: a meta-analysis of human neuroimaging studies. Neurosci Biobehav Rev 2018;94:76–92. 30067939 10.1016/j.neubiorev.2018.07.013 PMC6363360

[66] Vichaya EG Dantzer R. Inflammation-induced motivational changes: perspective gained by evaluating positive and negative valence systems. Curr Opin Behav Sci 2018;22:90–5. 29888301 10.1016/j.cobeha.2018.01.008 PMC5987547

[67] Iwata M Ota KT Duman RS. The inflammasome: pathways linking psychological stress, depression, and systemic illnesses. Brain Behav Immun 2013;31:105–14. 23261775 10.1016/j.bbi.2012.12.008 PMC4426992

[68] Verma V Sheikh Z Ahmed AS. Nociception and role of immune system in pain. Acta Neurol Belg 2015;115:213–20. 25547878 10.1007/s13760-014-0411-y

[69] Pavlov VA Tracey KJ. Neural circuitry and immunity. Immunol Res 2015;63:38–57. 26512000 10.1007/s12026-015-8718-1 PMC4743890

[70] Griep EN Boersma JW Lentjes EG. Function of the hypothalamic-pituitary-adrenal axis in patients with fibromyalgia and low back pain. J Rheumatol 1998;25:1374–81. 9676772

[71] Malatji BG Meyer H Mason S. A diagnostic biomarker profile for fibromyalgia syndrome based on an NMR metabolomics study of selected patients and controls. BMC Neurol 2017;17:88. 28490352 10.1186/s12883-017-0863-9 PMC5426044

[72] Bäckryd E Lind AL Thulin M. High levels of cerebrospinal fluid chemokines point to the presence of neuroinflammation in peripheral neuropathic pain: a cross-sectional study of 2 cohorts of patients compared with healthy controls. Pain 2017;158:2487–95. 28930774 10.1097/j.pain.0000000000001061 PMC5690569

