# Supplementary material for: Plasma protein patterns are strongly correlated with pressure pain thresholds in women with chronic widespread pain and in healthy controls—an exploratory case-control study
Source: Medicine (Baltimore). 2020 May 29;99(22):e20497. doi: 10.1097/MD.0000000000020497 (PMC12245271; doi:10.1097/MD.0000000000020497)
Supplement: SUPPLEMENTARY MATERIAL [file medi-99-e20497-s001.docx]

**Supplementary material**

***Proteins associated with low PPT in CWP***

There were four proteins associated with low PPT i.e. negatively correlated.

**Retinol-binding protein 4 (RBP4)**

RBP4 is the principal transport protein for retinol (vitamin A) in the circulation and considered as a proinflammatory adipokine. In agreement with the latter was found that RBP4 (spot no: 1013) had a negative correlation with PPT in CWP (Table 2). In trigeminal neuralgia increased levels of RBP4 were downregulated after microvascular decompression surgery ^1^. A preliminary study of serum proteome in FM patients reported elevated RBP4 ^2^. Involving activation of nuclear factor кB RBP4 induces inflammation in vascular endothelial cells by increasing expression of proinflammatory cytokines, chemokines and adhesion molecules ^3^.

**Apolipoprotein C-II**

This protein (spot no: 1051) correlated negatively with PPT in CWP. The same proteoform was also a significant regressor of psychological distress in the same cohort ^4^. A significantly increased plasma level was found in FM ^5^. Strong correlations exist between levels of triglycerides in blood and cardiovascular disease ^6^. Apolipoprotein C-II is found on triglyceride-rich lipo-proteins (TRL) and high-density lipoproteins. It is a cofactor of lipoprotein lipase, which is the main enzyme that hydrolyses plasma triglycerides on TRL ^6^. Both deficiency in and excess of apolipoprotein C-II are associated with hypertriglyceridemia ^7,8^. It has been suggested as cardiovascular disease risk factors ^7,9^.

**Complement factor B**

The complement system is part of the humoral response within the innate immune system ^10^. This system identifies pathogens that are not recognized by antibodies ^11^ and aid antibodies in clearing out damaged cells and microbes via the release of cytokines which in turn activate other cascades ^11^. There are three activation pathways: 1) classical activation pathway, 2) lectin pathway and 3) the alternative pathway. Complement factor B is an acute-phase protein and it is important for the complement activation via the alternative pathway ^12^.
This protein (spot no: 6832) correlated negatively with PPT. Another proteoform (spot no: 6902) was important for group separation CWP vs. CON ^13^ and between farmers with musculoskeletal disorders vs. controls ^12^; increased levels were found in subjects with pain. Another proteoform (spot no: 7901) was positively associated with psychological distress in the present CWP cohort ^4^. In major depression upregulated complement factor B levels have been found ^14^.

**Clusterin**

This protein (spot no: 114) correlated negatively with PPT in CWP and was also positively associated with psychological distress (four proteoforms spots nos: 114, 1104, 1113, 3214) in CWP ^4^. Clusterin attenuate inflammation and it is suggested as a biomarker in cancer, cardiovascular diseases, diabetes and metabolic syndrome ^15^. However, in healthy subjects clusterin plasma levels correlate with adiposity and systemic inflammation parameters and male sex ^16^. Lower serum levels have been found in hand osteoarthritis ^17^ and in degenerative scoliosis ^18^.

***Proteins positively associated with high PPT in CWP***

Two proteins correlated positively with PPT i.e. associated with high PPT values in PPT.

**Hemopexin**

Two proteoforms of hemopexin (spot nos: 5504 and 6608) were among the most important (positively correlated) regressors of PPT (Table 2); two other proteoforms of hemopexin (spot nos: 6501 and 5501) were also positive regressors. This is in agreement with the notion that hemopexin reduces inflammation and decreases oxidative stress ^19^. Hemopexin is multi-functional protectant against hemoglobin-derived heme toxicity as well as mitigating heme-mediated effects on immune cells, endothelial cells and stem cells that collectively contribute to driving inflammation, disturbing vascular hemostasis and blood-brain barrier function ^19,20^. Partly in contrast to the anti-inflammatory characteristics two proteoforms (spot nos: 4604 and 6608 (weakly significant)) showed positive correlations with psychological distress in the present CWP study^4^. Further, farmers with musculoskeletal disorders had increased plasma levels of two proteoforms of hemopexin ^12^.

**Secretory Immunoglobulin chain α**

Secretory Immunoglobulin chain α (spot no: 3717) correlated positively with PPT in CWP (Table 2). Two other proteoforms (spot nos: 4713 and 4807) were weak significant positive regressor of pain intensity in the same patient cohort ^4^. This agrees with the conclusion that secretory IgA (sIgA)can have both protective roles and pathophysiological roles. This protein is a heavy chain of IgA. The function of sIgA is to prevent passage of foreign substances into the circulation. IgA antibodies can be cleaved into Fc and Fab fragments and such autoantibodies may be associated with pain ^21^. In the saliva sIgA correlated positively with the healing process after wisdom teeth surgery ^22^. Hyperactive synovial IgA synthesis occur in several inflammatory joint diseases ^23^.

**The most important proteins for PPT in CON**

Among the significant proteins with high VIP (≥1.4) two showed positive associations with PPT and two negative correlations in CON.

***Proteins positively associated with PPT in CON***

**Complement C3 alpha chain**

Complement C3 is an essential molecule for all three pathways of the complement system ^10,24^. Complement C3 consist of two chains (alpha and beta). Complement C3 alpha chain (spot no: 6842) correlated positively with PPT in CON. Another proteoform correlated positively with age in CON ^4^.

**IG alpha-2 chain C region**

It represents 15-20 % of immunoglobulins in the blood. Upregulated plasma levels have been reported in colorectal cancer ^25^ and in type I and II diabetes ^26^. This protein was significantly downregulated in plasma from patients with chronic rheumatic mitral stenosis ^27^ and from patients with esophageal squamous cell carcinoma ^28^. It (spot no: 5511) showed a positive correlation with PPT in CON. A proteoform correlated negatively with age in CON ^4^. In CWP another proteoform was downregulated ^13^ and another proteoform was weakly but significantly and positively associated with psychological distress in CWP ^4^.

***Proteins negatively associated with PPT in CON***

**Alpha-2-macroglobulin**

Alpha-2-macroglobulin (spot no: 4902) correlated negatively with PPT in CON. Another proteoform correlated negatively with age in CON ^4^. Increased blood levels have been reported in deep venous thrombosis ^29^, in Alzheimer´s disease (AD) ^30^ and in type I diabetes ^26^. This acute-phase protein can inhibit a broad spectrum of proteases but it can also influence signaling of and bind cytokines and growth factors including neurotrophins (e.g., IL-6, platelet-derived growth factor, nerve growth factor (NGF), tumor-necrosis factor (TNF)-α, and IL-1β) ^31,32^. The anti-inflammatory effect of Alpha-2-macroglobulin is regulated by hypochlorite which is an oxidant generated during inflammation ^31^. It may have an important role in the interactions between cytokines and inflammatory processes ^32^.

**Vitamin D-binding protein**

Vitamin D-binding protein (spot no: 3408) was negatively associated with PPT in CON. This protein but another proteoform was upregulated in CWP ^13^ while farmers with pain had downregulated levels of this protein in plasma ^12^. A systematic review concluded that patients with endometriosis have higher levels of this protein than controls ^33^ but the literature is not in consensus. Vitamin D-binding protein is responsible for the transport of vitamin D and its hydroxylated metabolites in plasma but also has other metabolic roles e.g. transportation of fatty acids and endotoxin, and part of actin scavenging system and of the innate immunity ^34^.

# **References**

1. Farajzadeh A, Bathaie SZ, Arabkheradmand J, Ghodsi SM, Faghihzadeh S. Different Pain States of Trigeminal Neuralgia Make Significant Changes in the Plasma Proteome and Some Biochemical Parameters: a Preliminary Cohort Study. *J Mol Neurosci.* 2018;66(4):524-534.

2. Ruggiero V, Era B, Cacace E, et al. A preliminary study on serum proteomics in fibromyalgia syndrome. *Clin Chem Lab Med.* 2014;52(9):e207-210.

3. Du M, Martin A, Hays F, Johnson J, Farjo RA, Farjo KM. Serum retinol-binding protein-induced endothelial inflammation is mediated through the activation of toll-like receptor 4. *Mol Vis.* 2017;23:185-197.

4. Wåhlén K, Ghafouri B, Ghafouri N, Gerdle B. Plasma Protein Pattern Correlates With Pain Intensity and Psychological Distress in Women With Chronic Widespread Pain. *Front Psychol.* 2018;9:2400.

5. Ramirez-Tejero JA, Martinez-Lara E, Rus A, Camacho MV, Del Moral ML, Siles E. Insight into the biological pathways underlying fibromyalgia by a proteomic approach. *J Proteomics.* 2018;186:47-55.

6. Wolska A, Dunbar RL, Freeman LA, et al. Apolipoprotein C-II: New findings related to genetics, biochemistry, and role in triglyceride metabolism. *Atherosclerosis.* 2017;267:49-60.

7. Kei AA, Filippatos TD, Tsimihodimos V, Elisaf MS. A review of the role of apolipoprotein C-II in lipoprotein metabolism and cardiovascular disease. *Metabolism.* 2012;61(7):906-921.

8. Dallinga-Thie GM, Kroon J, Boren J, Chapman MJ. Triglyceride-Rich Lipoproteins and Remnants: Targets for Therapy? *Curr Cardiol Rep.* 2016;18(7):67.

9. Ooi EM, Barrett PH, Chan DC, Watts GF. Apolipoprotein C-III: understanding an emerging cardiovascular risk factor. *Clin Sci (Lond).* 2008;114(10):611-624.

10. Merle NS, Noe R, Halbwachs-Mecarelli L, Fremeaux-Bacchi V, Roumenina LT. Complement System Part II: Role in Immunity. *Front Immunol.* 2015;6:257.

11. Totsch SK, Sorge RE. Immune System Involvement in Specific Pain Conditions. *Mol Pain.* 2017;13:1744806917724559.

12. Ghafouri B, Carlsson A, Holmberg S, Thelin A, Tagesson C. Biomarkers of systemic inflammation in farmers with musculoskeletal disorders; a plasma proteomic study. *BMC Musculoskelet Disord.* 2016;17(1):206.

13. Wåhlén K, Olausson P, Carlsson A, Ghafouri N, Gerdle B, Ghafouri B. Systemic alterations in plasma proteins from women with chronic widespread pain compared to healthy controls: a proteomic study. *J Pain Res.* 2017;10:797-809.

14. Wang Q, Yu C, Shi S, et al. An analysis of plasma reveals proteins in the acute phase response pathway to be candidate diagnostic biomarkers for depression. *Psychiatry Res.* 2019;272:404-410.

15. Baralla A, Sotgiu E, Deiana M, et al. Plasma Clusterin and Lipid Profile: A Link with Aging and Cardiovascular Diseases in a Population with a Consistent Number of Centenarians. *PLoS One.* 2015;10(6):e0128029.

16. Won JC, Park CY, Oh SW, Lee ES, Youn BS, Kim MS. Plasma clusterin (ApoJ) levels are associated with adiposity and systemic inflammation. *PLoS One.* 2014;9(7):e103351.

17. Kropackova T, Sleglova O, Ruzickova O, Vencovsky J, Pavelka K, Senolt L. Lower serum clusterin levels in patients with erosive hand osteoarthritis are associated with more pain. *BMC Musculoskelet Disord.* 2018;19(1):264.

18. Zhu Y, Han S, Zhao H, et al. Comparative analysis of serum proteomes of degenerative scoliosis. *J Orthop Res.* 2011;29(12):1896-1903.

19. Montecinos L, Eskew JD, Smith A. What Is Next in This "Age" of Heme-Driven Pathology and Protection by Hemopexin? An Update and Links with Iron. *Pharmaceuticals (Basel).* 2019;12(4).

20. Ross AC. Impact of chronic and acute inflammation on extra- and intracellular iron homeostasis. *Am J Clin Nutr.* 2017.

21. Goebel A. Autoantibody pain. *Autoimmun Rev.* 2016;15(6):552-557.

22. Dostalova T, Kroulikova V, Podzimek S, Jelinkova H. Low-Level Laser Therapy After Wisdom Teeth Surgery: Evaluation of Immunologic Markers (Secretory Immunoglobulin A and Lysozyme Levels) and Thermographic Examination: Placebo Controlled Study. *Photomed Laser Surg.* 2017;35(11):616-621.

23. Elicabe RJ, Silva JE, Dave MN, et al. Association between IL-17 and IgA in the joints of patients with inflammatory arthropathies. *BMC Immunol.* 2017;18(1):8.

24. Silawal S, Triebel J, Bertsch T, Schulze-Tanzil G. Osteoarthritis and the Complement Cascade. *Clin Med Insights Arthritis Musculoskelet Disord.* 2018;11:1179544117751430.

25. Choi JW, Liu H, Shin DH, et al. Proteomic and cytokine plasma biomarkers for predicting progression from colorectal adenoma to carcinoma in human patients. *Proteomics.* 2013;13(15):2361-2374.

26. do Nascimento de Oliveira V, Lima-Neto ABM, van Tilburg MF, et al. Proteomic analysis to identify candidate biomarkers associated with type 1 diabetes. *Diabetes Metab Syndr Obes.* 2018;11:289-301.

27. Mukherjee S, Jagadeeshaprasad MG, Banerjee T, et al. Proteomic analysis of human plasma in chronic rheumatic mitral stenosis reveals proteins involved in the complement and coagulation cascade. *Clin Proteomics.* 2014;11(1):35.

28. Zhao J, Fan YX, Yang Y, et al. Identification of potential plasma biomarkers for esophageal squamous cell carcinoma by a proteomic method. *Int J Clin Exp Pathol.* 2015;8(2):1535-1544.

29. Lygirou V, Latosinska A, Makridakis M, et al. Plasma proteomic analysis reveals altered protein abundances in cardiovascular disease. *J Transl Med.* 2018;16(1):104.

30. Varma VR, Varma S, An Y, et al. Alpha-2 macroglobulin in Alzheimer's disease: a marker of neuronal injury through the RCAN1 pathway. *Molecular psychiatry.* 2017;22(1):13-23.

31. Cater JH, Wilson MR, Wyatt AR. Alpha-2-Macroglobulin, a Hypochlorite-Regulated Chaperone and Immune System Modulator. *Oxid Med Cell Longev.* 2019;2019:5410657.

32. Shimomura R, Nezu T, Hosomi N, et al. Alpha-2-macroglobulin as a Promising Biological Marker of Endothelial Function. *J Atheroscler Thromb.* 2018;25(4):350-358.

33. Sayegh L, Fuleihan Gel H, Nassar AH. Vitamin D in endometriosis: a causative or confounding factor? *Metabolism.* 2014;63(1):32-41.

34. Giampaolino P, Della Corte L, Foreste V, Bifulco G. Is there a relationship between Vitamin D and Endometriosis? An overview of literature. *Curr Pharm Des.* 2019.
